# Drug Repositioning via Graph Neural Networks: Identifying Novel JAK2 Inhibitors from FDA-Approved Drugs through Molecular Docking and Biological Validation

**DOI:** 10.3390/molecules29061363

**Published:** 2024-03-19

**Authors:** Muhammad Yasir, Jinyoung Park, Eun-Taek Han, Won Sun Park, Jin-Hee Han, Wanjoo Chun

**Affiliations:** 1 Department of Pharmacology, Kangwon National University School of Medicine, Chuncheon 24341, Republic of Korea; yasir.khokhar1999@gmail.com (M.Y.); jinyoung0326@kangwon.ac.kr (J.P.); 2 Department of Medical Environmental Biology and Tropical Medicine, Kangwon National University School of Medicine, Chuncheon 24341, Republic of Korea; ethan@kangwon.ac.kr (E.-T.H.); han.han@kangwon.ac.kr (J.-H.H.); 3 Department of Physiology, Kangwon National University School of Medicine, Chuncheon 24341, Republic of Korea; parkws@kangwon.ac.kr

**Keywords:** drug repositioning, Janus kinase 2 (JAK2), deep-learning, RDKit, DeepChem, GraphConvMol, graph convolutional neural network, molecular docking

## Abstract

The increasing utilization of artificial intelligence algorithms in drug development has proven to be highly efficient and effective. One area where deep learning-based approaches have made significant contributions is in drug repositioning, enabling the identification of new therapeutic applications for existing drugs. In the present study, a trained deep-learning model was employed to screen a library of FDA-approved drugs to discover novel inhibitors targeting JAK2. To accomplish this, reference datasets containing active and decoy compounds specific to JAK2 were obtained from the DUD-E database. RDKit, a cheminformatic toolkit, was utilized to extract molecular features from the compounds. The DeepChem framework’s GraphConvMol, based on graph convolutional network models, was applied to build a predictive model using the DUD-E datasets. Subsequently, the trained deep-learning model was used to predict the JAK2 inhibitory potential of FDA-approved drugs. Based on these predictions, ribociclib, topiroxostat, amodiaquine, and gefitinib were identified as potential JAK2 inhibitors. Notably, several known JAK2 inhibitors demonstrated high potential according to the prediction results, validating the reliability of our prediction model. To further validate these findings and confirm their JAK2 inhibitory activity, molecular docking experiments were conducted using tofacitinib—an FDA-approved drug for JAK2 inhibition. Experimental validation successfully confirmed our computational analysis results by demonstrating that these novel drugs exhibited comparable inhibitory activity against JAK2 compared to tofacitinib. In conclusion, our study highlights how deep learning models can significantly enhance virtual screening efforts in drug discovery by efficiently identifying potential candidates for specific targets such as JAK2. These newly discovered drugs hold promises as novel JAK2 inhibitors deserving further exploration and investigation.

## 1. Introduction

Drug repositioning involves identifying novel therapeutic uses for medications that have previously gained approval for different medical purposes [1]. It can notably accelerate the drug development process, enhance the utility of established drugs, and reveal novel treatments for ailments lacking effective remedies [2]. Accordingly, drug repurposing is becoming an increasingly important area of research in drug development. Computer-aided drug design (CADD) has become an essential tool in the domain of drug discovery and development [3]. Utilizing computational algorithms and software, CADD enables efficient screening of large compound libraries, offering a faster and more cost-effective alternative to traditional experimental approaches [4]. A primary strength of CADD is its capacity to swiftly assess a large number of compounds, minimizing the extensive laboratory testing in traditional experimental studies, which can be time consuming and expensive [5]. 

Artificial intelligence is rapidly expanding and possesses significant promise in transforming the drug development process [6]. Deep learning (DL), a subset of artificial intelligence, enables its models to assimilate data and formulate predictions or decisions without explicit programming [7]. DL plays a pivotal role in drug development by analyzing vast datasets encompassing genetic and clinical data. This analysis aids in discovering new drug targets, predicting drug effectiveness with accuracy, and fine-tuning drugs [8,9]. One of its primary advantages is the capability to analyze large and complex datasets [10]. Whereas traditional data analysis methods, like manual examination and statistical techniques, can be labor intensive and time consuming, DL models offer swift and adept data analysis, discerning patterns and forecasting outcomes, which in turn fast-tracks the drug development process [11]. An additional strength of DL in drug development is its capability to predict the potency and toxicity of compounds [12]. By analyzing extensive datasets, DL models can discern trends suggesting drug effectiveness and potential toxicity, enabling the prediction of these attributes before the synthesis and laboratory evaluation. Therefore, integrating DL within CADD can markedly improve the speed, efficiency, and success of the drug discovery, making it significant in drug discovery and development.

Janus kinases (JAKs) belong to a family of non-receptor tyrosine kinases crucial for cellular signaling, especially within the immune system [13,14,15]. Disruption in JAK function is associated with various inflammatory disorders, such as rheumatoid arthritis, psoriasis, and inflammatory bowel disease [16,17]. Four main members constitute the JAK family: JAK1, JAK2, JAK3, and TYK2 [18]. Each has unique traits and distinct cellular roles. Specifically, JAK1 is associated with signaling via the interferon-α receptor, while JAK3 primarily operates through the common gamma chain receptor [19,20,21]. JAK2, however, interfaces with a multitude of cytokines, including erythropoietin, thrombopoietin, and interleukin-6 [14,22], positioning it with a more expansive signaling capability compared to JAK1 and JAK3. Notably, JAK2’s involvement has been identified in conditions like polycythemia vera, essential thrombocythemia, and myelofibrosis [23]. While certain JAK1 and JAK3 mutations are reported in acute lymphoblastic leukemia [24], JAK2’s role appears more central in the onset of diverse diseases [25,26,27]. Given this context, our study focuses on the development of novel JAK2 inhibitors. In this study, we employed a graph neural network algorithm to train on datasets containing active and decoy JAK2 inhibitors. Subsequently, we screened an FDA-approved drug library to identify potential JAK2 inhibitors for drug repurposing. We further assessed the selected compounds using molecular docking techniques and their biological activity was validated using a JAK2 kinase assay kit to discover novel JAK2 inhibitors.

## 2. Results and Discussions

**Figure 1:**
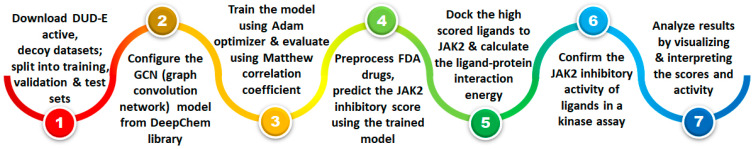
The process of integrating deep-learning, molecular docking, and experimental evaluation for drug repurposing novel JAK2 inhibitors.

The process of integrating deep-learning, molecular docking, and experimental evaluation for drug repurposing of novel JAK2 inhibitors is illustrated in Figure 1. The process comprised seven distinct phases: (1) data acquisition and preparation from the DUD-E database, (2) configuration of the graph convolutional network model, (3) training and evaluation of the deep learning model, (4) predictive assessment of FDA-approved drugs, (5) molecular docking for the top-predicted drugs, (6) experimental validation of potential candidates through JAK2 kinase assay, and (7) analysis of results to confirm the validity of repurposing FDA-approved drugs as novel JAK2 inhibitors.

### 2.1. JAK2 Active and Decoy Datasets and Its Preprocessing Using RDKit

**Figure 2:**
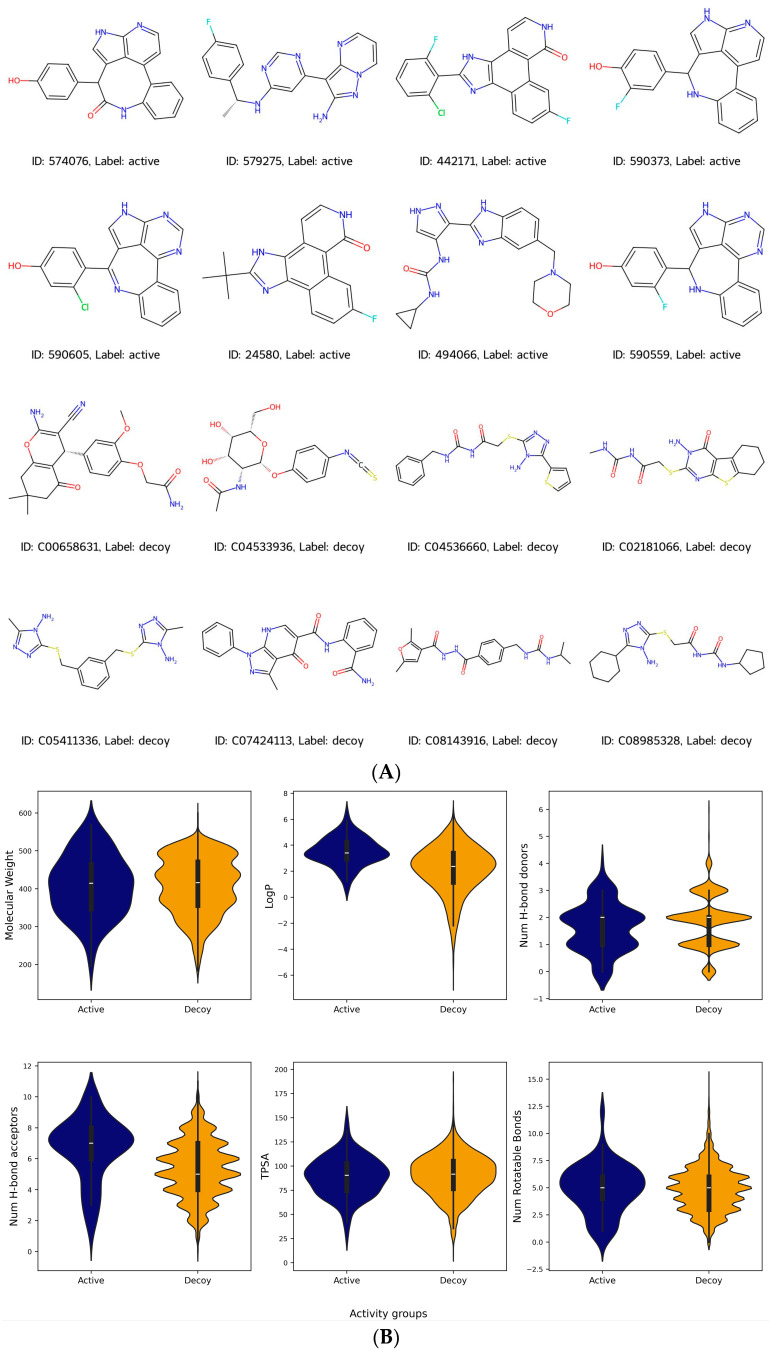
(**A**) Representative image of active and decoy compounds. (**B**) Distribution of molecular weight, LogP, number of hydrogen bond donors/acceptors, TPSA, and number of rotatable bonds in active and decoy compounds.

The DUD-E (Database of Useful Decoys: Enhanced) database is an open-access database that hosts benchmark sets of protein–ligand complexes. It encompasses a set of experimentally confirmed active compounds, their affinities against diverse targets, and associated decoys that are confirmed not to bind with the target. Though these decoys share similar physicochemical properties with the active compounds, their two-dimensional topology differ [28]. The DUD-E database has frequently served as a benchmark for the creation and evaluation of computational docking techniques [29,30]. The JAK2 dataset in the DUD-E database (https://dude.docking.org/targets/JAK2) (accessed on 15 January 2024) features 107 active compounds, curated from an initial set of 246 compounds, paired with 6500 decoy compounds. Figure 2A provides illustrative images of the structures of both active and decoy compounds, with labels in the legend to distinguish them. To evaluate the physicochemical distinctions between active and decoy compounds, we used RDKit (Version 2023.09.6), a free chemoinformatics software toolkit, to calculate their molecular attributes. Upon comparison, we observed minimal variations in the distribution patterns of molecular features such as weight, LogP, the number of hydrogen bond donors/acceptors, topological polar surface area (TPSA), and number of rotatable bonds (Figure 2B).

### 2.2. Deep-Learning Model Setup, Training, and Evaluation

**Figure 3:**
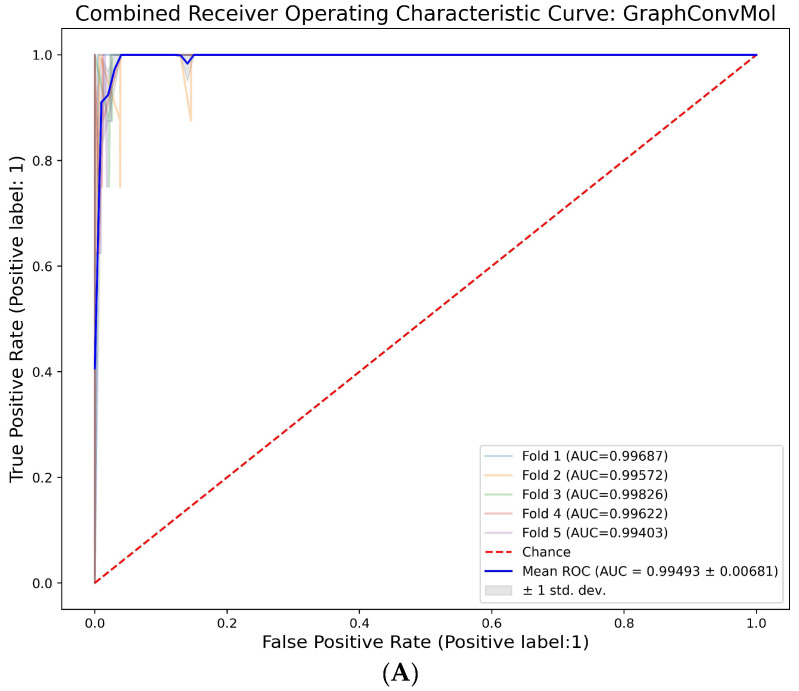

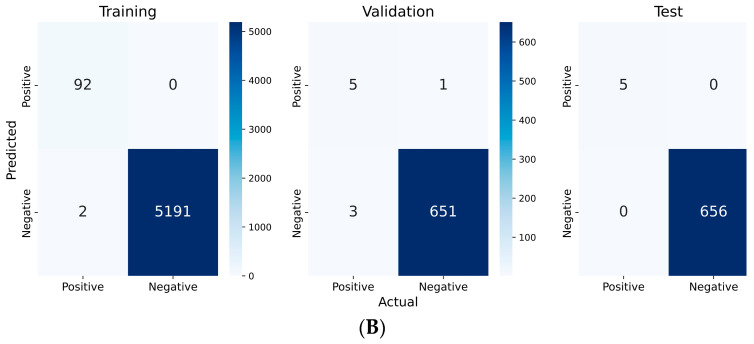
(**A**) The AUR-ROC curve of five-fold cross validation of the training dataset. (**B**) The confusion matrix values of training, validation, and test datasets.

DeepChem is an open-source Python library designed for deep learning applications within drug discovery and cheminformatics. It offers a comprehensive suite of tools for managing molecular data and harnessing various deep learning techniques for tasks like molecular attribute forecasting, virtual ligand screening, and molecule optimization [31,32]. In this research, we employed the GraphConvMol model from DeepChem to discern differences between active and decoy compounds within the JAK2 dataset. This model, an integral part of the DeepChem suite, uses a form of graph convolutional neural network to process molecular graphs, turning them into fixed-size representation vectors. Each atom is denoted as a node, and covalent bonds become edges in this molecular graph. The algorithm involves a series of message-passing phases, during which each atom communicates its unique features to adjacent atoms. After collecting messages from neighboring atoms, the data are synthesized to update the current atom’s attributes. The final representation of the molecule is formulated by combining the individual atom representations and further refining them through feed-forward neural networks. As GraphConvMol facilitates the end-to-end learning of molecular structures, it stands as a robust asset in cheminformatics endeavors, specifically in predicting molecular properties and drug discovery [33,34]. The JAK2 dataset was split into training, validation, and test sets at a ratio of 8:1:1, and then subjected to the GraphConvMol model using cross-validation with a fold of 5. To assess the model’s performance, the AUC (Area Under the Curve) of the ROC (Receiver Operating Characteristic) curve was computed for the training, validation, and test datasets. The ROC curve, generated from a five-fold cross-validation on the training dataset, illustrated a True Positive Rate (TPR) value of 1 at an exceptionally low False Positive Rate (FPR) with an AUC value of 0.992 (Figure 3A). This suggests that the GraphConvMol model exhibits high sensitivity in identifying positive instances while effectively minimizing false positives.

To evaluate the performance of GraphConvMol on DUD-E datasets, metrics such as precision, recall, F1 score, sensitivity, accuracy, and specificity were calculated across training, validation, and test datasets (Table 1). The training dataset showed reliable performances, with only 2 out of 94 positive instances misclassified as negative (recall: 0.98). In the validation dataset, there was one false positive out of 652 negatives (precision: 0.83) and 3 false negatives out of 8 instances (recall: 0.63). The lower performance metrics in the validation dataset may be due to the limited number of active compounds. However, the model demonstrated optimal performance in the test dataset, achieving a score of 1 in all metrics (Table 1). 

Due to the disproportionate number of decoys relative to active compounds in the dataset, the Matthews correlation coefficient (MCC) was utilized to assess the performance of the GraphConvMol model. This metric is particularly effective for datasets with such imbalances. The averaged MCC values from five-fold cross-validation processes were 0.96 for the training set and 0.76 for the validation set. A perfect prediction accuracy is indicated by an MCC of 1, highlighting that those scores of 0.96 and 0.76 demonstrate the model’s robustness and dependability. It is generally expected for the MCC value of the test set to surpass that of the validation set since the model, after being trained on the training set, is then tested on the novel and unencountered data of the validation set. Furthermore, the variation in MCC values observed across the five-fold cross-validation suggests that the model is not overly fitted to the training data.

### 2.3. Prediction of JAK2 Inhibitory Potential from FDA-Approved Drugs

**Figure 4:**
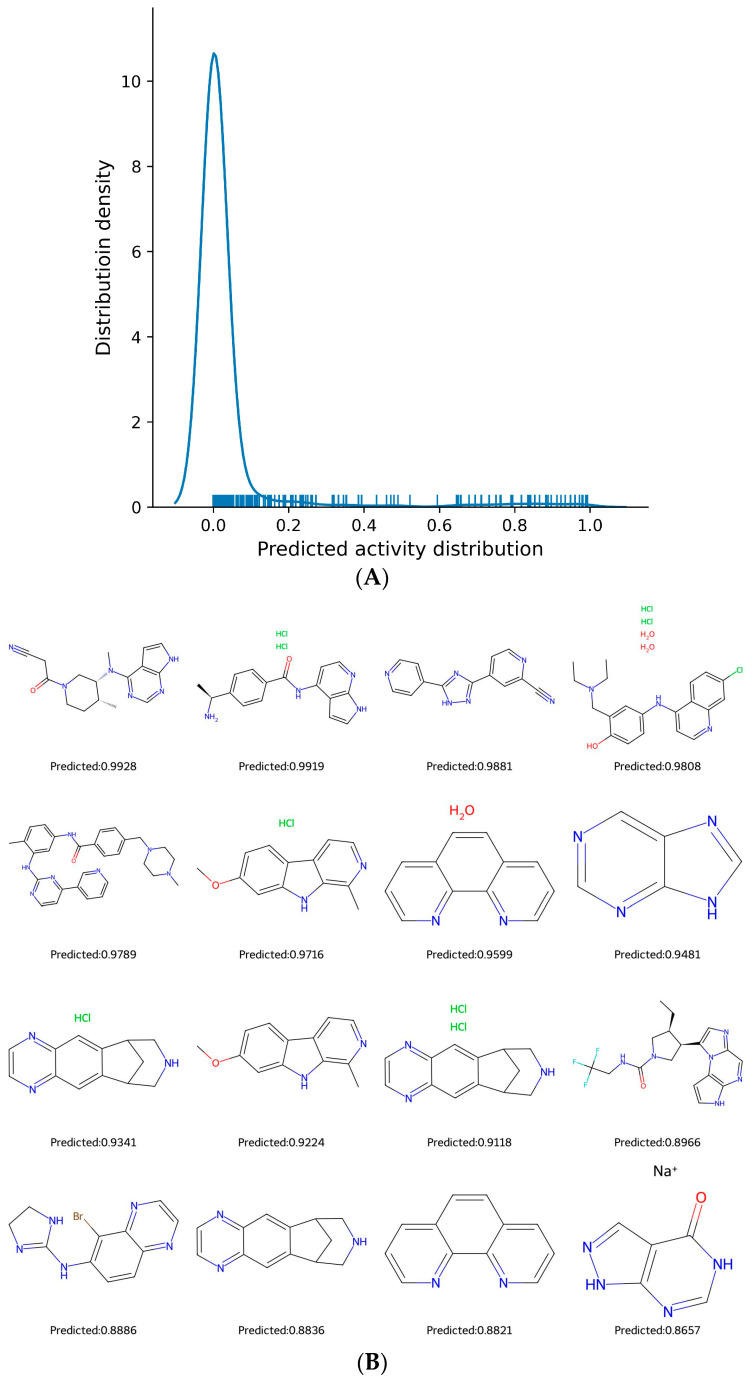
(**A**) Distribution of GraphConvMol prediction. (**B)** Structures of highly predicted compounds from FDA-approved drugs.

Repositioning FDA-approved drugs offers distinct advantages. Given that these drugs have already undergone rigorous pre-clinical and clinical evaluations for safety, dosage, and pharmacokinetics, their repositioning often means shorter development periods, reduced costs, and a higher probability of success. The trained model, utilizing the GraphConvMol algorithm from DeepChem, processed SMILES strings of FDA-approved drugs to assess their potential for JAK2 inhibitory activity. Predictions on JAK2 inhibitory capability for these drugs spanned a range from 0 (inactive) to 1 (highly active). While a majority of the compounds were deemed inactive, a small subset was identified as potential actives (Figure 4A). Figure 4B presents structures of select compounds that were predicted to have high activity, with labels showcasing their anticipated values.

Noticeably, several of top-ranked compounds such as ruxolitinib, baricitinib, tofacitinib, and upadacitinib (listed in Table 2) are well-known JAK2 inhibitors. This strongly indicates the high robustness and reliability of the present model. From the set of drugs highly predicted by the GraphConvMol model, we selected several candidates for further evaluation regarding their potential JAK2 inhibitory actions through molecular docking and experimental assessment. Gefitinib, a tyrosine kinase inhibitor used in acute lymphoblastic leukemia [35], ribociclib, a CDK kinase inhibitor employed in the treatment of metastatic breast cancer [36], amodiaquine, an inhibitor of heme polymerase inhibitor used for malaria [37], and topiroxostat, an inhibitor of xanthine oxide used for gout [38], were among the chosen drugs. These drugs have not been previously reported to be associated with JAK2 inhibition.

### 2.4. Structural Analysis of the JAK2 Protein

**Figure 5:**
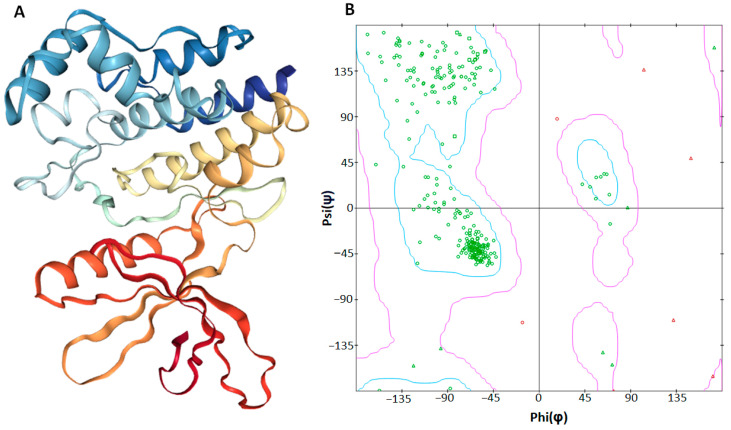
(**A**,**B**). Three-dimensional structure (**A**) of the JAK2 protein and the computed Ramachandran plot (**B**), calculated by discovery studio.

A non-receptor tyrosine kinase JAK2 belongs to the Janus kinase family and has been linked to signaling by the single chain receptors (Epo-R, Tpo-R, GH-R, and PRL-R), the GM-CSF receptor family’s (IL-3R, IL-5R, and GM-CSF-R), and the type II cytokine receptor family’s (interferon receptor) [39]. It was constructed by 311 amino acids forming a single chain (PDBID 3JY9). Loops, α-helices, and β-sheets are present in the overall structure of JAK2 (Figure 4). Furthermore, a VADAR 1.8 structural assessment demonstrated that JAK2 was constructed by 40% α-helices, 22% β-sheets, 37% coils, and 23% turns. Moreover, the Ramachandran plots analysis revealed that 95.1% of amino acids occur in the favored region, while 98.6% of residues were in the allowed zone of dihedral angles phi (φ) and psi (ψ) (Figure 5B). 

### 2.5. The Binding Pocket Analysis

**Figure 6:**
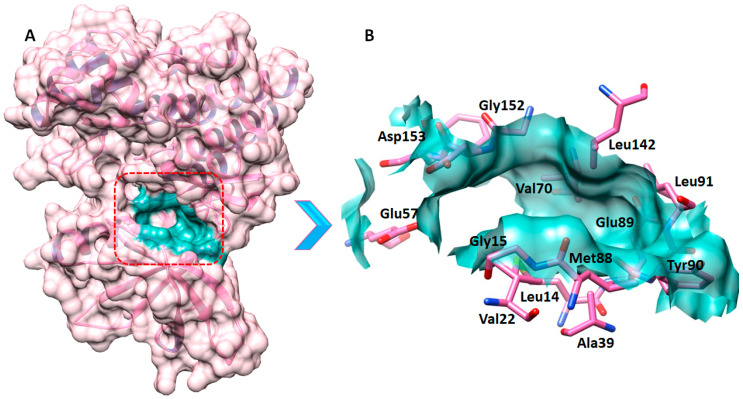
(**A**,**B**). The figure (**A**) manifests the full structural representation and the binding pocket of JAK2. The whole protein is colored as hot pink, the interior helixes are colored dark slate blue, while the binding surface area is colored as light sea green. Furthermore, the active site residues are mentioned on their position in the active region of the target protein in black (**B**).

Along with its structure and position inside a protein, a binding pocket’s function is influenced by the group of amino acid residues that surround it [40]. Using the Discovery Studio ligand interaction method, the binding pocket residues of JAK2 were obtained from the interaction of JAK2 and co-crystalized ligand and mentioned as Leu14, Gly15, Val22, Ala39, Leu142, Glu57, Val70, Met88, Tyr90, Leu91, Gly152, and Asp153. Therefore, the co-crystalized ligand was chosen by the current selection approach to define the CDocker binding sphere. Furthermore, the binding sphere was subjected to contraction to limit it to the accurate position respective to our selected binding pocket residues. The binding sphere values were X = 12, Y = 13, Z = 2.6, and the radius value was fixed as 7.8 to study the interaction of selected compounds in the active region of JAK2 (Figure 6A,B).

### 2.6. Molecular Docking Analysis

The top 20 screened compounds were docked against JAK2. The docked complexes were evaluated and examined independently and scored based on the minimal docking energy and interaction energy values. The Discovery Studio CDocker module forecasts two types of energy values (CDocker energy and CDocker interaction energy). The terms CDocker energy and CDocker interaction energy are used to describe the energy involved in the various interactions between the ligand and the receptor. CDocker energy displays the overall docking energy based on the 3D structural and physiochemical features of the ligand and protein, whereas the strength and nature of each individual contact between the ligand and the receptor are revealed by CDocker interaction energy. It calculates how much the overall binding strength is affected by intermolecular forces such Van der Waals forces, electrostatic interactions, and hydrogen bonds [41,42,43]. The top 20 docking results concerning the CDocker interaction energy score were depicted in Table 3. Therefore, ribociclib demonstrate the lowest interaction energy values. Moreover, the gefitinib and amodiaquine came up in the top 10 docked compounds, although they exhibit a high CDocker interaction energy as compared to ribociclib, they exhibit a lower interaction energy than the reference compound tofacitinib (gefitinib, amodiaquine, and tofacitinib manifest −50.6 kcal/mol, −44.4 kcal/mol, and −40.0 kcal/mol, respectively). Topiroxostat comparatively revealed a high interaction energy (−28.8 kcal/mol) compared to the reference compound.

### 2.7. Binding Interaction Analysis against JAK2

The top 20 screened compounds that were docked against the JAK2 protein were further analyzed by Discovery studio and UCSF Chimera to examine and confirm the binding interaction of ligands with the active site amino acid residues of JAK2.

**Figure 7:**
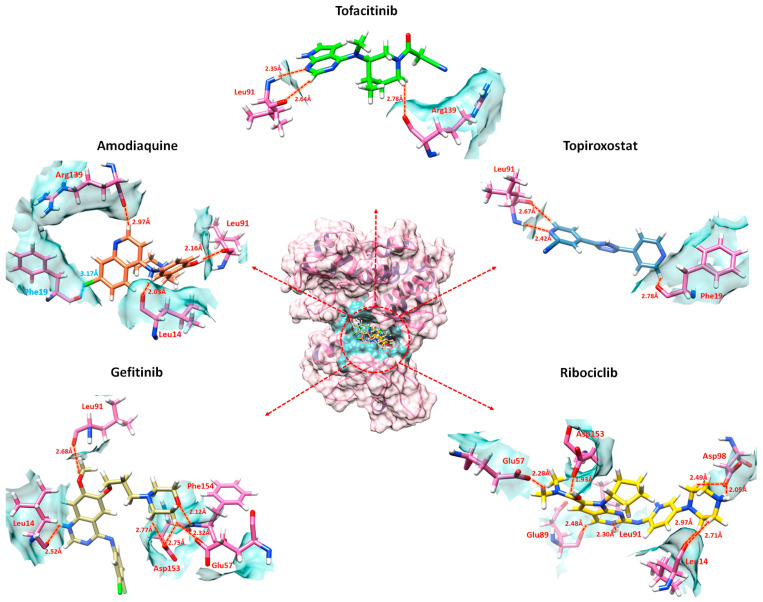
The graphical representation of combined amodiaquine, topiroxostat, gefitinib and ribociclib interaction in comparison with tofacitinib against the active region amino acid residues of JAK2. The JAK2 protein is represented in the center (hot pink) while the interactions of ligands are predicted in different dimensions. Each ligand is colored differently in the active pocket of JAK2 (amodiaquine: coral, topiroxostat: steel blue, gefitinib: dark khaki, ribociclib: gold). The hydrogen bonds, bonding distance and bonding amino acid residues are colored red while the other interacting amino acid residues are colored black. Furthermore, the halogen bond is depicted in cyan color.

Ribociclib compounds, which manifest the lowest interaction energy molecular docking energy, manifest the strongest interaction against JAK2 (Figure 7). The ribociclib-JAK2 docked complex expressed eight hydrogen bonds which include the residues Glu57, Asp153, Glu89, Leu91, Leu14, and Asp98. Two oxygen atoms of ribociclib form hydrogen bonds against Glu57 and Asp153 with a bond length of 2.28 Å and 1.93 Å, respectively. Another two oxygen atoms of ligand exhibit two hydrogen bonds with the same Asp98 with a bonding distance of 2.49 Å and 2.05 Å. Moreover, the other two oxygen atoms also formed two hydrogen bonds with the same Leu14 with a bonding distance of 2.97 Å and 2.71 Å. Another solo oxygen atom of ribociclib revealed a hydrogen bond with Glu89 with a bond length of 2.48 Å. Furthermore, a nitrogen atom of ligand expresses a hydrogen bond against Leu91 with a bonding distance of 2.30 Å. 

The ligand–protein docking analysis of Amodiaquine showed that the ligand binds within the active region of the target protein as shown in Figure 7. The Amodiaquine-Jak2 docked complex exhibits three hydrogen bonds and one halogen bond. A halogen bond is formed when there is evidence of a net attractive interaction between an electrophilic region associated with a halogen atom in one chemical entity and a nucleophilic region in another or the same molecular entity [44]. The hydrogen atom of Amodiaquine formed a hydrogen bond with Arg139 with a bonding distance of 2.97 Å. Additionally, two other hydrogen atoms of ligand formed hydrogen bonds with Leu91 and Leu14 with bond lengths of 2.16 Å and 2.03 Å, respectively. Furthermore, the chlorine atom of ligand formed a halogen bond with Phe19 with a bonding distance of 3.17 Å. Topiroxostat was confined in the active binding pocket of the JAK2 protein and formed three hydrogen bonds with active region amino acid residues (Figure 7). The topiroxostat-JAK2 docked complex showed a hydrogen atom of formed hydrogen bonds with Leu91 with a bond length of 2.67 Å. Furthermore, a nitrogen atom of topiroxostat also formed a hydrogen bond with Leu91 with a bond length of 2.42 Å. Moreover, another hydrogen atom of ligand formed a hydrogen bond with Phe19 with a bonding distance of 2.78 Å.

The ligand–protein docking analysis of tofacitinib showed that ligands become docked within the active region of the target protein, as shown in Figure 7. The tofacitinib-JAK2 docked complex forms three hydrogen bonds which include the residues Lue91 and Arg139. The oxygen atom of tofacitinib forms a hydrogen bond against Leu91 with a bond length of 2.64 Å. Furthermore, the nitrogen atom of the ligand also forms a hydrogen bond with Leu91 with a bonding distance of 2.35 Å. Moreover, the oxygen atom of ligand exhibits a hydrogen bond against Arg139 with a bonding distance of 2.78 Å. The gefitinib compound also manifests high interactions following ribociclib. The ribociclib-JAK2 docked complex exhibit six hydrogen bonds (Figure 7). The oxygen atom of ligand formed a hydrogen bond with Asp154 with bond length of 2.12 Å. An oxygen atom of ligand revealed two hydrogen bonds with the same Asp153 with bond length of 2.32 Å and 2.75 Å. Moreover, the other two oxygen atoms of gefitinib showed two hydrogen atoms with the same Leu91 with the bond length of 2.68 Å and 2.33 Å. Furthermore, an oxygen atom of ligand revealed a hydrogen bond against Leu14 with a bonding distance of 2.52 Å.

These interactions strongly suggest that the predicted drugs block the active region of JAK2 by hindering with the active region amino acid residues. 

### 2.8. Experimental Validation

**Figure 8:**
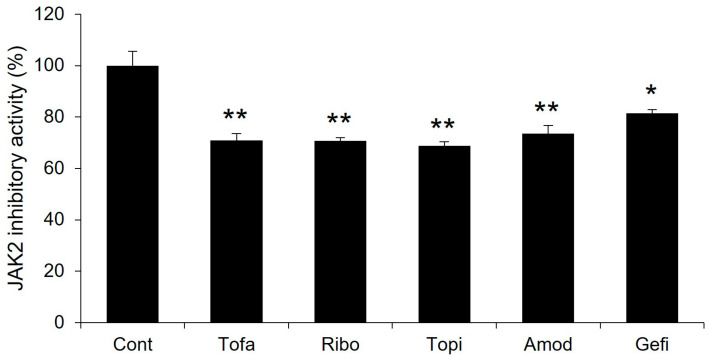
JAK2 inhibitory activity of highly predicted JAK2 inhibitors in comparison to tofacitinib. Single (*) and double (**) marks represent statistical significance at *p* < 0.05 and *p* < 0.01, respectively.

JAK2 inhibitory activity of highly predicted drugs and tofacitinib, a reference drug, was experimentally evaluated using a JAK2 kinase assay kit. Both tofacitinib and the other drugs exhibited significant inhibition of the JAK2 enzymatic activity at 25 nM. This concentration is consistent with the previously documented IC_50_ values for the inhibitory activity of tofacitinib against JAK2 [45]. Remarkably, each of the test drugs demonstrated significant JAK2 inhibition, with their effectiveness closely paralleling that of tofacitinib (Figure 8). This suggests that these drugs hold promise as potential novel JAK2 inhibitors.

### 2.9. Structural Evaluation and Similarity Comparison

**Figure 9:**
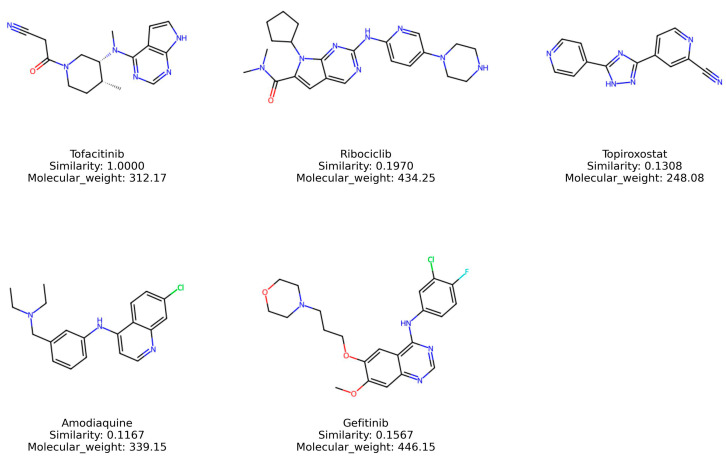
Structures of highly predicted JAK2 inhibitors.

To evaluate the structural similarity among the top-ranked drugs, the Tanimoto similarity measure in RDKit was utilized. Tofacitinib and several top-ranked drugs in JAK2 inhibitory potential prediction exhibit structural characteristics. Each of these drugs incorporates one or more heterocyclic rings along with aromatic moieties (Figure 9). Further, these compounds possess diverse substituents attached to their primary scaffolds, which likely influence their interactions with JAK2 proteins. However, despite these structural motifs, an assessment using the Tanimoto similarity coefficient showed that their overall structural similarity was not notably high (Table 4). In general, while no exact threshold exists for defining similarity, a Tanimoto similarity value below 0.5 is often regarded as indicative of dissimilarity in a range from 0 to 1. On this scale, a value of 0 denotes no similarity at all, and a value of 1 represents complete similarity.

**Figure 10:**
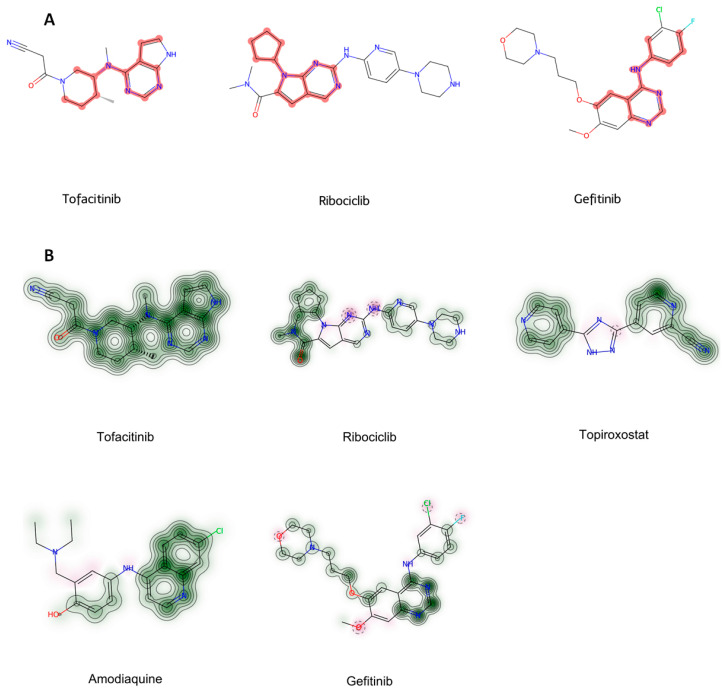
(**A**,**B**). Graphical representation of common structural motif found with Maximum Common Substructure (MCS) (**A**) and similarity maps (**B**).

While the top-ranked drugs exhibited limited overall similarity to tofacitinib, it is still possible that these drugs share specific structural features. To explore this, the Maximum Common Substructure (MCS) algorithm in RDKit was applied. Tofacitinib and the four top-ranked drugs were analyzed using the MCS algorithm in RDKit with the threshold of 0.5. This analysis grouped tofacitinib, ribociclib, and gefitinib together, with their common substructures highlighted in red color (Figure 10A). This result implies that factors other than the structural motif, such as the spatial arrangement of specific conformations, might contribute to the inhibitory activity on JAK2 protein. Furthermore, similarity maps using fingerprints in RDKit were employed to illustrate whether the top-ranked drugs possessed the structural motif of tofacitinib (Figure 10B). The similarity maps of the top-ranked drugs revealed the presence of structural motif of tofacitinib in their chemical structures. These findings from the MCS and similarity map findings provide valuable information to guide further optimization of the selected compounds.

The highly predicted compounds, including tofacitinib, ribociclib, topiroxostat, amodiaquine, and gefitinib, are characterized by their LogP, solubility, gastrointestinal (GI) absorption, blood–brain barrier (BBB) permeation, CYP2D6 inhibition, and Lipinski violation (Table 5). Notably, tofacitinib exhibits moderate lipophilicity and solubility with high GI absorption but lacks BBB permeation. Ribociclib and topiroxostat, despite their high GI absorption, demonstrate contrasting BBB permeation abilities, with ribociclib showing the potential inhibition of CYP2D6. Amodiaquine and gefitinib, with high lipophilicity, solubility, and GI absorption, showcase BBB permeation and CYP2D6 inhibition. These data provide a comprehensive overview of the ADME profiles, aiding in the assessment of these compounds’ potential suitability for drug development.

## 3. Methodology

### 3.1. JAK2 Datasets and FDA-Approved Drug Library

JAK2 active and decoy datasets were obtained from the DUD-E website (https://dude.docking.org/) (accessed on 15 January 2024). The active dataset contained 107 compounds, while the decoy dataset had 6500 compounds. All molecules were expressed as canonicalized SMILES strings with DUD-E ID and ChEMBL ID numbers. Compounds were labeled as active and decoy in legend. The FDA-approved drug library was downloaded from the website of Selleck Chemicals (https://www.selleckchem.com) (accessed on 16 January 2024). FDA-approved drug molecules, totaling 3105 in number, were represented in SDF (structure-data file) format and transformed into SMILES strings using RDKit. 

### 3.2. Molecular Descriptor Generation Using RDKit

Molecular descriptors for the compounds were generated using RDKit. RDKit is an open-source, high-performance cheminformatics and machine learning toolkit written in Python (https://www.rdkit.org) (accessed on 20 January 2024). The toolkit offers features for calculating molecular descriptors, producing chemical attributes, and visualizing chemical data.

### 3.3. Deep Learning Architecture

The JAK2 active and decoy datasets were split for training, validation, and test sets in 8:1:1 ratio. The GraphConvMol model from DeepChem (https://deepchem.io/models) (accessed on 22 January 2024) was employed as the deep learning algorithm. The GraphConvMol, being a graph convolutional neural network, adeptly processes graph-structured inputs like molecular graphs. A concise overview of its architecture is as follows: Initially, the molecular structures are transformed into graphs where atoms represent nodes and bonds acting as edges. Following this, several graph convolutional layers are employed to derive hierarchical features from these molecular graphs. These layers are equipped with adaptable parameters that have varying weights, fine-tuning the model’s learning to precisely grasp the nuances of molecular structures. During the training phase, the model refines its performance by minimizing a loss function in relation to the input molecular datasets. This optimization adjusts the convolutional layers’ weights through backpropagation. Ultimately, the model seeks to predict specific attributes of molecules, such as solubility, bioactivity, and potential toxicity, grounded on their structures.

### 3.4. JAK2 Structure Retrieval

The X-ray structure of human JAK2 protein (PDB ID: 3JY9 with 2.10Å resolution) was obtained from the Protein Data Bank (PDB) (https://www.rcsb.org) (accessed on 25 January 2024), and minimized Discovery studio and UCSF Chimera [46,47]. The JAK2 protein, made up of helices, sheets, coils, and turns, was subjected to further analysis like quantitative protein structural analysis using the online freely accessible server VADAR 1.8 (http://vadar.wishartlab.com/) (accessed on 25 January 2024). Additionally, Discovery Studio was employed to analyze and compute the Ramachandran graphs [46]. 

### 3.5. Prediction of Active Binding Site

The interacting site in the protein’s holo-structure most likely determines the binding pocket of the protein where the active ligand binds [48]. The JAK2 X-ray structure was retrieved from PDB (PDB ID: 3JY9). The co-crystalized ligand was selected and the binding sphere was constructed by the current selection technique in the binding site window of Discovery Studio to define the active pocket. The interacting amino acids were chosen by the ligand interaction approach of Discovery Studio for the accuracy of the binding site generation. Consequently, the binding sphere was contracted to become restricted to our selected amino acids. 

### 3.6. Molecular Docking

Molecular docking is the most commonly used method for the evaluation of the interactions and conformations of ligands against the target proteins [49]. It anticipates the association strength or binding compatibility between ligand and protein based on preferred orientation by using scoring algorithms [40,50]. The waters and the ligand molecule were removed from the receptor and the hydrogens were added by Discovery Studio’s protein preparation module, prior to docking. The ligand preparations were also carried out for reference and candidate compounds, tautomerization was carried out, ionization was changed, and bad valences were fixed by Discovery Studio’s ligand preparation module. Furthermore, the conformation prediction was to the top 10. Therefore, the Discovery Studio’s CDocker module was employed to perform molecular docking of the screened ligands against JAK2 with the default orientation and conformation. The lowest CDocker interaction energy values (in kcal/mol) were utilized to estimate the best-docked complexes.

### 3.7. Binding Interaction Analysis

The 3D graphical evaluations were carried for the docked complexes using UCSF Chimera 1.10.1 [47] and Discovery Studio to study the interactions of screened drugs against JAK2 protein. 

### 3.8. JAK2 Kinase Inhibitory Activity Assay

Tofacitinib, topiroxostat, and gefitinib were obtained from Sigma (St. Louis, MO, USA), and ribociclib and amodiaquine were obtained from Selleck Chemicals (Houston, TX, USA). The compounds were dissolved in DMSO. JAK2 kinase activity was measured using the JAK2 Assay Kit from BPS Bioscience (#79520, San Diego, CA, USA) following the manufacturer’s instructions. The reactions were incubated at 30 degrees Celsius for 45 min. Then, 50 µL of the Kinase-Glo MAX reagent (Promega, Madison, WI, USA, #V6071) was added and covered the plate with aluminum foil, and incubated at room temperature for 15 min. Finally, luminescence measurements of the ATP product were obtained using a microplate spectrophotometer (Molecular Devices, San Jose, CA, USA). All assays were performed in triplicate.

### 3.9. Statistical Analysis

All values shown in the figures were expressed as the mean ± SD obtained from at least three independent experiments. Statistical significance was analyzed by two-tailed Student’s *t*-test. Data with values of *p* < 0.05 were considered as statistically significant. 

## 4. Conclusions

As the landscape of drug development evolves, becoming more intricate and expensive, it is imperative to leverage cutting-edge techniques that streamline the process. The integration of artificial intelligence into this process offers a fast-track approach to pinpointing potential candidate compounds that might be the next therapeutic breakthroughs. The research outlined in this study underscores the compelling advantages of such a strategy and its efficiency in drug discovery. This study innovates drug discovery by integrating graph convolutional networks (GCN) with molecular docking, surpassing traditional methods. GCN captures complex three-dimensional molecular structures, enhancing predictive accuracy for binding affinities. Combined with molecular docking, it offers a more comprehensive screening, efficiently identifying potential drug candidates. It marks a significant step forward in drug screening, potentially applicable to a wide range of molecular targets. By deploying the graph neural network algorithm within the DeepChem library’s deep learning module, we identified compounds that efficiently fit the active region of the target JAK2, effectively obstructing its active site at a computational level. Several of the top predicted drugs are recognized JAK2 inhibitors, attesting to the solidity of our methodology. Additionally, several compounds, including ribociclib, amodiaquine, topiroxostat, and gefitinib, previously not linked with JAK2 inhibition, exhibited a promising JAK2 inhibitory potential. Experimental validation confirmed the deep learning and molecular docking results. As a result, we propose these compounds as prospective novel JAK2 inhibitors. In conclusion, a deep learning-centric approach to drug repositioning emerges as a pivotal strategy in advancing drug discovery, not just for JAK2 inhibitors but for a broad spectrum of therapeutic targets.

## 5. Limitations

In this study, the datasets were primarily derived from FDA-approved drugs and the DUD-E database. While these sources are valuable, they may not fully represent the extensive diversity of molecular structures, which could impact the generalizability of our model. Consequently, the performance of our model might vary when applied to datasets with different chemical spaces, potentially limiting its broader applicability. Future research directions will focus on incorporating a wider range of chemical libraries to enhance dataset diversity. Additionally, we plan to explore advanced computational algorithms to address potential biases in the data and improve the robustness of our model. These steps are crucial for adapting our methodology to other protein targets and assessing its utility across diverse therapeutic areas.

## Figures and Tables

**Table 1:** Performance metrics of GraphConvMol model.

	Precision	Recall	F1 score	Accuracy	Specificity
**Training**	1.000	0.979	0.989	0.999	1.000
**Validation**	0.833	0.625	0.714	0.994	0.999
**Test**	1.000	1.000	1.000	1.000	1.000

Precision = True Positives/(True Positives + False Positives); recall = True Positives/(True Positives + False Negatives); F1 score = 2 × ((precision × recall)/(precision + recall)); accuracy = (True Positives + True Negatives)/total population; specificity = True Negatives/(True Negatives + False Positives).

**Table 2:** Detailed information of drugs that were predicted with high JAK2 inhibitory potential.

Smiles	Neg	Pos	Name	Target	Use
N#CC[C@H](C1CCCC1)n1ccc2ncnc3[nH]ccc23)cn1	0.0002	0.9998	Ruxolitinib	JAK Inhibitor	Myelofibrosis, Anti cancer drug
COc1cc2ncnc(Nc3ccc(F)c(Cl)c3)c2cc1OCCCN1CCOCC1.Cl	0.0004	0.9996	Gefitinib	Tyrosine Kinase, EGFR inhibitor	Non-small cell lung carcinoma
Nc1ncnc2[nH]cnc12	0.0007	0.9993	Adenine	Nucleobase	Nucleotide
c1ncc2nc[nH]c2n1	0.0007	0.9993	Purine	Heterocyclic aromatic organic compound	DNA and RNA formation
CC[C@H](Nc1ncnc2[nH]cnc12)c1nc2cccc(F)c2c(=O)n1-c1ccccc1	0.0011	0.9989	Idelalisib	Phosphoinositide 3-kinase inhibitor	Blood cancer
C#Cc1cccc(Nc2ncnc3cc4c(cc23)OCCOCCOCCO4)c1	0.0021	0.9979	Icotinib	Epidermal growth factor receptor tyrosine kinase inhibitor (EGFR-TKI)	Non-small cell lung cancer
c1coc(CNc2ncnc3nc[nH]c23)c1	0.0031	0.9969	Kinetin	Proapoptotic anti-proliferative plant growth regulator	Cell division
Cl.O=C(O)c1cn(-c2ccc(F)cc2)c2cc(N3CCNCC3)c(F)cc2c1=O	0.0031	0.9969	Sarafloxcin	Quinolone antibiotic drug	Antibiotic
CCS(=O)(=O)N1CC(CC#N)(n2cc(-c3ncnc4[nH]ccc34)cn2)C1	0.0033	0.9967	Baricitinib	JAK2 inhibitor	Rheumatoid arthritis
C[C@@H]1CCN(C(=O)CC#N)C[C@@H]1N(C)c1ncnc2[nH]ccc12	0.0033	0.9967	Tofacitinib	JAKs inhibitor	Rheumatoid arthritis
Nc1ncnc2c1ncn2[C@@H]1O[C@H](CO)[C@@H](O)[C@@H]1O.O	0.0039	0.9961	Vidarabine	Human herpesvirus 1 DNA polymerase	Antiviral
Nc1ncnc2c1ncn2[C@H]1C[C@H](O)[C@@H](CO)O1.O	0.0039	0.9961	2′-Deoxyadenosine	Phosphodiesterase inhibitor	Energy source
CN(C)C(=O)c1cc2cnc(Nc3ccc(N4CCNCC4)cn3)nc2n1C1CCCC1	0.0045	0.9955	Ribociclib	CDK4/CDK6 kinase inhibitor	Metastatic breast cancer
CCN(CC)CCCC(C)Nc1ccnc2cc(Cl)ccc12	0.0051	0.9949	Chloroquine	Heme polymerase inhibitor	Malaria, Rheumatoid arthritis
COc1cc2nc(N3CCN(C(=O)C4CCCO4)CC3)nc(N)c2cc1OC.Cl.O.O	0.0074	0.9926	Terazosin	Alpha 1-adrenergic receptor inhibitor	Adrenaline blocker
CCN(CC)Cc1cc(Nc2ccnc3cc(Cl)ccc23)ccc1O.Cl.Cl.O.O	0.0077	0.9923	Amodiaquine	Heme polymerase inhibitor	Malaria
C[C@H](Nc1ncnc2[nH]cnc12)c1cc2cccc(Cl)c2c(=O)n1-c1ccccc1	0.0081	0.9919	Duvelisib	PI3K inhibitor	Chronic lymphocytic leukemia
CC[C@@H]1CN(C(=O)NCC(F)(F)F)C[C@@H]1c1cnc2cnc3[nH]ccc3n12	0.0082	0.9918	Upadacitinib	JAK inhibitor	Rheumatoid arthritis
c1cnc2c(c1)ccc1cccnc12	0.0086	0.9914	1,10-Phenanthroline	Fe(II) chelator	Metal chelator
C[C@H](Cn1cnc2c(N)ncnc21)OCP(=O)(O)O.O	0.0100	0.9900	Tenofovir	Nucleotide reverse transcriptase inhibitor	HIV
Cc1cc(/C=C/C#N)cc(C)c1Nc1ccnc(Nc2ccc(C#N)cc2)n1	0.0117	0.9883	Rilpivirine	Transcriptase inhibitor	HIV
C#Cc1cccc(Nc2ncnc3cc(OCCOC)c(OCCOC)cc23)c1	0.0122	0.9878	Erlotinib	Tyrosine kinase, EGFR inhibitor	Non-small cell lung cancer (NSCLC), pancreatic cancer
Cc1ccc(NC(=O)c2ccc(CN3CCN(C)CC3)cc2)cc1Nc1nccc(-c2cccnc2)n1	0.0172	0.9828	Imatinib	Tyrosine kinase, Bcr-abl inhibitor	Chronic myeloid leukemia
N#Cc1cc(-c2n[nH]c(-c3ccncc3)n2)ccn1	0.0177	0.9823	Topiroxostat	Xanthine oxidase inhibitor	Hyperuricemia (gout)
O=c1[nH]cnc2[nH]ncc12.[Na+]	0.0196	0.9804	Allopurinol	Xanthine oxidase inhibitor	Hyperuricemia (gout)
O.S=c1nc[nH]c2nc[nH]c12	0.0298	0.9702	6-Mercaptopurine	Purine nucleotide synthesis inhibitor	Antimetabolite, Antineoplastic
C=C[C@H]1CN2CC[C@H]1C[C@H]2[C@H](O)c1ccnc2ccc(OC)cc12.Cl.O.O	0.0301	0.9699	Quinine	Potassium channel blocker	Antimalarial, Analgesic
Cl.Cl.c1cnc2cc3c(cc2n1)C1CNCC3C1	0.0337	0.9663	Varenicline	Nicotinic receptor blocker	Smoking cessation
O=c1[nH]cnc2nc[nH]c12	0.0365	0.9635	Hypoxanthine	Nucleic acid synthesis	Malaria parasite cultures
CN[C@@H]1C[C@H]2O[C@@](C)([C@@H]1OC)n1c3ccccc3c3c4c(c5c6ccccc6n2c5c31)C(=O)NC4	0.0414	0.9586	Staurosporine	PKC inhibitor	Cancer

The term ‘Neg’ refers to non-active outcomes, while ‘Pos’ indicates active outcomes. The predictive values are quantified where a value of 1 represents a perfect prediction, and a value of 0 signifies no possibility of the predicted outcome.

**Table 3:** The docking energy values (kcal/mol) of top 20 screened docked FDA compounds against JAK2 protein, calculated by Discovery Studio.

Compounds	Cdocker Interaction Energy (kcal/mol)	CDocker Energy (kcal/mol)
Ribociclib	−58.0	−5.3
Imatinib	−52.6	−24.3
Staurosporine	−52.2	100.1
Gefitinib	−50.6	−15.5
Adiphenine	−47.5	−32.4
Difloxacin	−47.2	−26.1
Amodiaquine	−44.4	−19.8
Naratriptan	−42.5	−31.8
Y-33075	−42.0	−31.1
Rilpivirine	−41.3	−31.8
Tofacitinib	−40.0	−29.3
Dibucaine	−39.8	−17.6
Amsacrine	−39.6	−8.2
Chloroprocaine	−33.4	−21.5
Topiroxostat	−28.8	−22.6
Pinacidil	−28.6	−20.6
Varenicline	−25.6	19.9
Phenanthroline	−23.0	−4.8
Pargyline	−22.5	−18.9
Allopurinol	−18.6	−4.0

**Table 4:** Tanimoto similarity comparison of highly predicted JAK2 inhibitors.

Similarity	Tofacitinib	Ribociclib	Topiroxostat	Amodiaquine	Gefitinib
Tofacitinib	-	0.196970	0.130841	0.114754	0.156716
Ribociclib	0.196970	-	0.146154	0.146853	0.180645
Topiroxostat	0.130841	0.146154	-	0.186916	0.131783
Amodiaquine	0.114754	0.146853	0.186916	-	0.257812
Gefitinib	0.156716	0.180645	0.131783	0.257812	-

**Table 5:** ADME properties of highly predicted JAK2 inhibitors.

Name	LogP	Solubility	GI Absorption	BBB Permeation	CYP2D6 Inhibition	Lipinski Violation
Tofacitinib	1.22	−3.34	High	No	No	0
Ribociclib	2.12	−5.51	High	No	Yes	0
Topiroxostat	1.38	−5.24	High	No	Yes	0
Amodiaquine	4.6	−8.18	High	Yes	Yes	0
Gefitinib	3.92	−7.94	High	Yes	Yes	0

## Data Availability

The data that support the findings of this study are available from the corresponding author upon reasonable request.
